# The secret of health in daily cuisine: typical healthy vegetables in local markets in central Myanmar

**DOI:** 10.1186/s13002-020-00425-w

**Published:** 2020-11-25

**Authors:** Yu Zhang, Jian-Wen Li, Myint Myint San, Cory William Whitney, Thae Thae San, Xue-Fei Yang, Aye Mya Mon, Pyae Phyo Hein

**Affiliations:** 1 Key Laboratory of Economic Plants and Biotechnology, Kunming Institute of Botany, Chinese Academy of Sciences, Kunming, China; 2 Southeast Asia Biodiversity Research Institute, Chinese Academy of Sciences, Nay Pyi Taw, Myanmar; 3 Yunnan Key Laboratory for Wild Plant Resources, Kunming Institute of Botany, Chinese Academy of Sciences, Kunming, China; 4 Forest Research Institute, Ministry of Natural Resources and Environmental Conservation, Nay Pyi Taw, Myanmar; 5 University of Bonn, Bonn, Germany; 6 University of Chinese Academy of Sciences, Beijing, China

**Keywords:** Central Myanmar, Healthy Vegetables, Market, Bamar People, Traditional Myanmar Medicine

## Abstract

**Background:**

Central Myanmar is located in the Indo-Burma biodiversity hotspot, and the Bamar people are the main ethnic group, which settled there over 1000 years ago. Despite being the core region of the country, central Myanmar has been ignored in previous ethnobotanical studies. Local healthy foods and knowledge are regarded as treasures for resource development and pharmaceutical drug discovery, and market surveys are a good strategy in ethnobotanical research. Thus, we collected and documented typical vegetables and local knowledge in local markets and then analysed the diversity and local knowledge of these vegetables.

**Materials and methods:**

Observations and interviews were used in the field study, and 10 markets and fairs were selected in central Myanmar. A total of 277 vegetable stalls or shops were visited. We compared the local knowledge we collected with selected important and typical herbal books on traditional Myanmar medicine. Quantitative analysis, including frequency of citation (FC), relative frequency of citation (RFC) and use value (UV), was used to assess the diversity and local knowledge of these vegetables.

**Results:**

A total of 132 plant taxa from 47 botanical families and 116 genera were collected. Most (106 taxa, 80.3%) of these vegetables were cited by the informants as functional foods that had health benefits, while others were regarded as merely “good for health”. The main health function of the vegetables was treating digestive problems. Sixty-four species were recorded in selected herbal books on traditional Myanmar medicine, and forty-seven taxa were not recorded in these books but were nonetheless used as healthy vegetables by local people. Twenty-eight species of vegetables were collected from wild places.

**Conclusion:**

The diversity and local knowledge of healthy vegetables in central Myanmar were rich. Nevertheless, the diversity of wild vegetables was seemingly relatively low. The possible reason was that we counted only the vegetables that were from entirely wild sources as “wild vegetables”. The most frequently cited vegetables were commonly cultivated species, which reflects the fact that plants cultivated on a large scale comprise the major source of vegetables. Some lesser known vegetables could reflect the unique food culture of local people, but most of these were cited only a few times by the interviewees, which caused low UV and RFC rankings for them in the league table. In addition, future research should pay more attention to the food safety of these vegetables.

**Supplementary Information:**

The online version contains supplementary material available at 10.1186/s13002-020-00425-w.

## Background

Local knowledge of health is a treasure for resource development and pharmaceutical drug discovery. Healthy foods, especially healthy vegetables, are important resources for local people in maintaining health [1], especially in Asian countries [2–7]. Local people have abundant knowledge of the collection, preparation, cooking, cultivation, edible safety, health function, and resource management of vegetables [8]. According to our previous studies, healthy vegetables have abundant functional compounds [9]. In addition, healthy vegetables are important sources of micro-nutrients and vitamins [10, 11]. Therefore, these vegetables have enormous potential for health-related research and industries such as drug discovery, food nutritional engineering and pharmaceuticals. However, most local knowledge is known only to native people or transmitted in small and limited areas. Therefore, such knowledge can easily disappear over time [8]. Moreover, global change and biodiversity loss may lead to the disappearance of local knowledge [12]. Hence, it is necessary to scientifically collect, document, analyse and explain local knowledge of healthy vegetables. Many studies about local healthy foods have been published in mainstream ethnobiology, ethnopharmacology and alternative medicine journals. Nevertheless, for some less-developed countries or remote poor regions, there are still few related studies.

Myanmar, which lies between China and India, is well known for its rich biodiversity and cultural diversity. As one of the key regions designated as global hotspots for biodiversity protection, Myanmar has an abundance of plant resources [13]. The U.S. National Herbarium, the Forest Department of Myanmar and the University of Yangon provided a Myanmar plant checklist containing over 3000 species [14]. KM Lwin and MKT Lwin catalogued a list of medicinal plants in Myanmar that contained 1500 species [15], and Defilipps et al. [16] recently reviewed the main information of 472 Myanmar medicinal plant species. For thousands of years, Buddhism has been widely distributed in Myanmar and has impacted almost every aspect of life there, including medicine. Vinaya, one of the Tripitaka philosophies of Buddhism about the regulation of life, is the theoretical basis of Myanmar traditional medicine. In Buddhist Vinaya practices, a qualified Buddhist follows strict dietary rules to maintain the health of both the body and spirit. Unhealthy dietary habits and bad foods can cause serious digestive problems, which are detrimental to Buddhist practice [3]. Therefore, in addition to medicine, Myanmar people use food to maintain health and treat ailments by following Vinaya in the traditional lifestyle; a well-known Myanmar proverb states that “medicine is food, food is medicine”. However, there are still few studies on this subject in international journals and books. The only related studies published in English have shown rich local medicinal knowledge among ethnic minority communities in Chin State [17], Shan State [18], Kachin State [19] and Mon State [20]. Nevertheless, the core region of Myanmar, central Myanmar and its main ethnic population, the Bamar people, have been ignored in previous ethnobotanical studies.

Market surveys are considered a good strategy for the preliminary screening of potential ethnopharmacological plant resources [9, 21]. According to related previous studies, markets are important for glimpsing the diversity and trade structure of the medicinal flora of a country or region [22–24]. Markets also reflect the public health and epidemic diseases of local communities [25]. Participating in markets is an important way for people to exchange goods, information and knowledge. As “showing stages” of local knowledge, local markets have been the focus of many researchers [26]. Local food markets are systems for food material and information exchange that deliver health, economic, environmental and social benefits to local communities [27]. Therefore, local food markets are ideal places to research the diversity and use knowledge of local vegetables [28–31]. In local markets, wild vegetables have often been the focus of ethnobotanical studies because of the close relationship between wild vegetable diversity and local wild species biodiversity [21, 32–38].

With funding from the National Science Foundation of China and Southeast Asia Biodiversity Research Institute, Chinese Academy of Sciences (SEABRI-CAS) and the support of the Myanmar Forest Research Institute (FRI), we designed and carried out the present study. In our preliminary investigation, we found that there was great diversity of vegetables in the local food markets of Myanmar [9]. This raised the following questions: (1) How many species of local typical vegetables were sold in these markets? (2) How many of these species were regarded as healthy vegetables by local people? (3) Which species were also recorded as medicinal plants in available traditional Myanmar medicine herbal books, and was the local health knowledge of plants similar to the records in the traditional Myanmar medicine literature and books?

Therefore, in this paper, our main work included (1) surveying the local markets and then recording, collecting, identifying and cataloguing the typical vegetables; (2) interviewing local people, documenting their knowledge of the health benefits of the collected vegetables, and then analysing the information about the vegetables and local knowledge; and (3) comparing the local knowledge we collected with the traditional medicine literature.

## Materials and Methods

### Study area

Central Myanmar is widely recognized for its history and ancient civilization. Central Myanmar was the birthplace of the flourishing Myanmar culture, and almost all of the capitals of Myanmar historical dynasties, such as Sri-ksetra, Hanthawaddy, Bagan, Bago, Taungoo, Inwa, Shwebo, Amarapura and Mandalay, were founded in central Myanmar [39, 40]. Most of the native residents of central Myanmar are Bamar people, the main ethnic group that settled in Myanmar over 1000 years ago. Most of the Bamar people are Theravada Buddhists, and Buddhist beliefs deeply affect the lifestyles of the Bamar people [40].

Generally, central Myanmar is classified as a tropical wet and dry climate zone. Due to the rain shadow of the Arakan Mountains, a large part of central Myanmar has a hot, semi-arid climate. The quite dry climate limits the development of forest in central Myanmar, and most of the region is covered by sparse open vegetation similar to savanna [39]. However, nourished by the Ayeyarwady River, the region is characterized by fertile soil and sufficient irrigation water, which causes it to become the most important agricultural district of Myanmar [40].

### Field survey and data collection

**Fig. 1:**
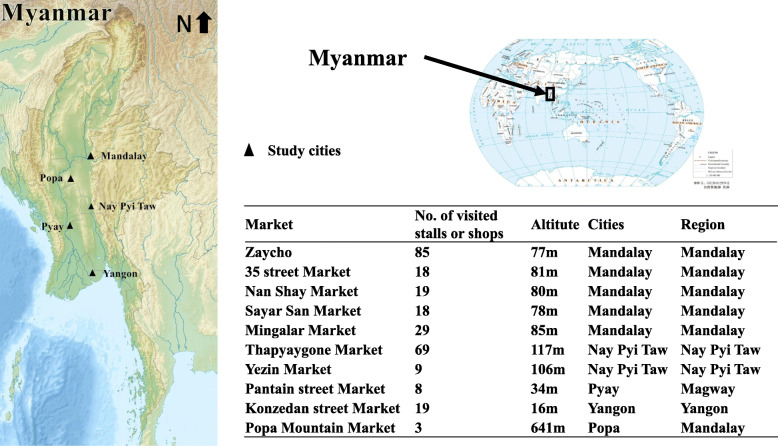
The study sites and markets

We carried out the field survey in 10 regular markets of the Mandalay Region, Magway Region, Yangon Region and Nay Pyi Taw Union Territory (Fig. 1), representing the typical food markets of central Myanmar. Because Mandalay is regarded as the centre of Myanmar culture, half of these markets were in Mandalay. We visited these markets in different seasons from 2015 to 2018, and we visited each market at least twice in 1 year. Moreover, the data from the markets in Mandalay and Nay Pyi Taw Union Territory were collected every month from 2016 to 2017.

**Table 1:**
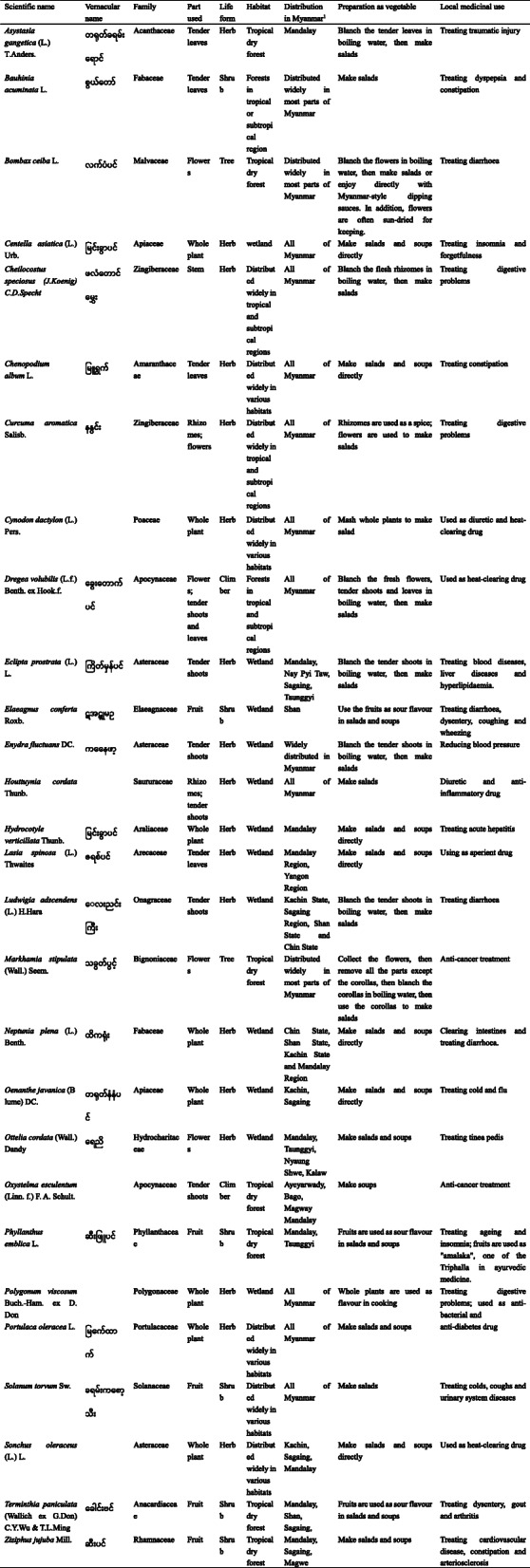
Wild vegetables

^a^Information from Kress et al. [14]

In total, 277 vegetable stalls or shops were randomly selected and investigated, and plant species found in these stalls and shops were recorded. Vegetables that were not largely cultivated worldwide were the major focus of our survey.

In the present study, we considered a “vegetable” a plant or plant product that was used for dishes in daily cuisine. Healthy vegetables are vegetables that local people consider to have health benefits.

The frequency of occurrence of each vegetable was investigated and evaluated using the following methods. First, we surveyed the target market quickly and counted the number of shops or stalls with numeric counters for the purpose of knowing the sizes and number of shops or stalls to establish the number of randomly selected shops or stalls in each target market. Second, we used digital cameras to record full images of the vegetables sold at each randomly selected shop or stall. Finally, we recorded the species found at each shop or stall by reviewing the photos. Then, we calculated the frequency of occurrence of each species. Meanwhile, the owners of these shops or stalls were set as the informants for participatory observation and semi-structured interviews.

Participatory observation and semi-structured interviews were conducted with the informed consent of the respondents to gather knowledge on the health benefits of vegetables. Methods of preparation, function and amount of usage for were recorded for species with health benefits. We recorded the habitat and source information of each vegetable. The interviews were carried out in Burmese and then translated into English with the facilitation of Myanmar collaborators from the Forest Research Institute (FRI). The informants were mainly vegetable sellers in the markets.

### Species identification

Voucher specimens of species were prepared and identified with the help of a specialist at the Kunming Institute of Botany, Chinese Academy of Sciences, China, and at the FRI. Voucher specimens were collected for most species (70%) and deposited at the Herbarium of the Myanmar Forest Research Institute (RAF) and the Herbarium of the Kunming Institute of Botany (KUN) (Additional file 1, Appendix A); the exceptions were common vegetables such as cabbage, tomato and eggplant. The identification was based on “A Checklist of the Trees, Shrubs, Herbs, and Climbers of Myanmar” [14] and the flora of surrounding areas such as the flora of China [41] and of Yunnan [42]. The scientific names were checked using a service provided by The Plant List [43].

**Table 2:**
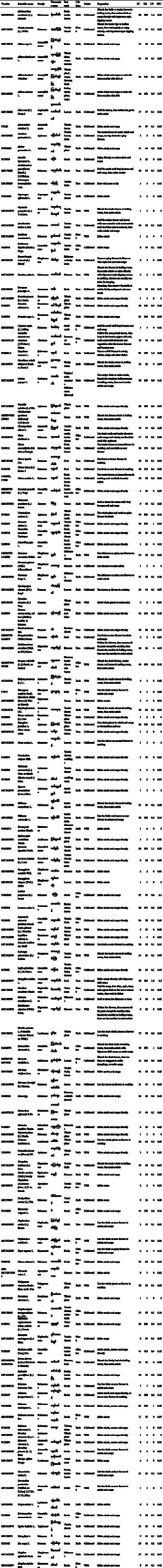
Plant list

### Categorizing the functions and conservation status of vegetables with health benefits

In traditional and local (folk) medicinal systems, the descriptions of ailments and diseases are usually diverse, which may cause difficulties in analysis. Thus, it was necessary to normalize the information with common standards before analysis. In the present study, all ailments were categorized with the classification of the patient’s reason for the encounter (CPRE) from the WHO (http://www.who.int/classifications/icd/adaptations/icpc2/en/). The species conservation status was checked with the IUCN Red List version 2019-2 [44].

### Quantitative ethnobotany analysis

Data from the field survey were organized in an Excel sheet for quantitative analysis. The use reports (URs) were prepared following Tardío and Pardo-de-Santayana [45] and further used to calculate quantitative ethnobotany indices: frequency of citation (FC), relative frequency of citation (RFC) and use value (UV). In the present study, quantitative analysis was used to assess the importance of the plants in the markets.

Frequency of citation (FC) was calculated as the sum of informants who cited a use for a particular species. Relative frequency of citation (RFC) was used to show the importance of each species in the study area [45]. The RFC values were calculated according to the following formula:

$$ RFC=\frac{FC_s}{N} $$

where FC_*s*_ is frequency of citation and *N* is the total number of informants in the survey. A high RFC value for a species indicates that the species was used both frequently and by a high proportion of informants in the study area.

Use value (UV) was used to measure the relative importance of species used locally [46]. The UV was calculated according to the following formula:

$$ UV=\frac{\sum {U}_i}{n} $$

where *Ui* is the number of URs cited by each informant for a given species and *n* refers to the total number of informants. A high UV indicates that a species is important and used frequently in the study area.

### Bibliographic comparison

Information on health benefits was compared with the available literature on traditional Myanmar medicine and modern phytochemical and pharmacological research. The traditional Myanmar medicine references were selected for important typical herbal books on traditional Myanmar medicine, including Defilipps and Krupnick (2018) [16], Kyaw Soe and Tin Myo Ngwe (2014) [47] and Ministry of Health Department of Traditional Medicine of Myanmar (2000) [48]. These books were recommended by Professor Kyaw Thein Htay, former president of the University of Traditional Myanmar Medicine in Mandalay, and his colleagues. These books include most of the medicinal plant species used in mainstream traditional Myanmar medicine.

## Results

### Diversity and parts used of the collected vegetable species

**Fig. 2:**
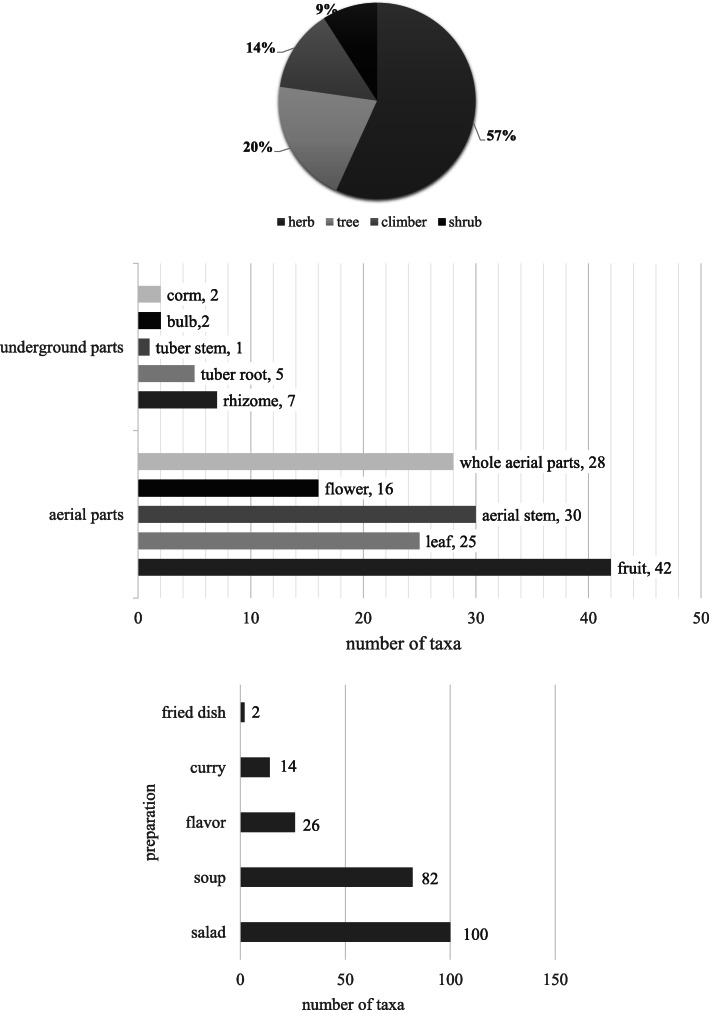
The diversity of plants and uses of vegetables. **a** Life forms of the vegetables. **b** Taxa frequencies of use parts of the vegetables. **c** Taxa frequencies of the preparation methods of the vegetables

In total, 132 plant taxa were collected. Two were identified at the genus level, 127 at the species level, two at the subspecies level and one as a variant, and they belonged to 47 botanical families and 116 genera (Additional file 1, Appendix A). The most commonly found family was Fabaceae (17 species), followed by Cucurbitaceae (10 species). Most genera (105) contained one species, while only 10 genera contained more than two species. The genera with the most species were *Allium* (four species) and *Citrus* (four species). More than half (75 taxa) were herbaceous plants, followed by trees (27 species), climbers (18 taxa) and shrubs (12 taxa) (Fig. 2a).

The plant parts used were fruits (42 taxa), leaves (25 taxa), aerial stems (30 taxa), flowers (16 taxa), whole aerial parts (28 taxa) and underground parts (17 taxa in total), including rhizomes, tuber roots, tuber stems, bulbs and corms (Fig. 2b). The recorded vegetables were prepared and used as salads (100 taxa), curries (14 taxa), soups (82 taxa), fried dishes (2 taxa) and flavouring (26 taxa) (Fig. 2c).

### The most popular vegetables

In total, 2361 URs were obtained from 277 informants. Twenty-five taxa had more than 100 URs, while nine had just one UR. The top five species with the highest UR and UV values were mango (*Mangifera indica*, UR = 504, UV = 1.819), chili (*Capsicum annuum*, UR = 455, UV = 1.643), okra (*Abelmoschus esculentus*, UR = 380, UV = 1.372), water spinach (*Ipomoea aquatica*, FC = 310, UV = 1.119) and cucumber (*Cucumis sativus*, UR = 273, UV = 0.986). The RFC results were slightly different from the FC and UV results. Chili, eggplant (*Solanum melongena*) and *Brassica rapa* (RFC = 0.329) had the highest RFC values, followed by tea leaf (*Camellia sinensis* var. *assamica*, RFC = 0.271), water spinach (RFC = 0.227) and okra (RFC = 0.209).

### Wild vegetables

Ethnobotanical studies on vegetables often include wild vegetables. In the present study, we considered “wild vegetables” to be vegetables that were not cultivated but collected from the wild environment. Twenty-eight species are recorded (Table 1). Among them, *Dregea volubilis* (UR = 230) was the species most frequently seen in the markets, followed by *Centella asiatica* (UR = 105). These species were also recorded as medicinal plants in the selected herbal books on traditional Myanmar medicine [16, 47, 48].

### Health functions and medicinal uses

**Fig. 3:**
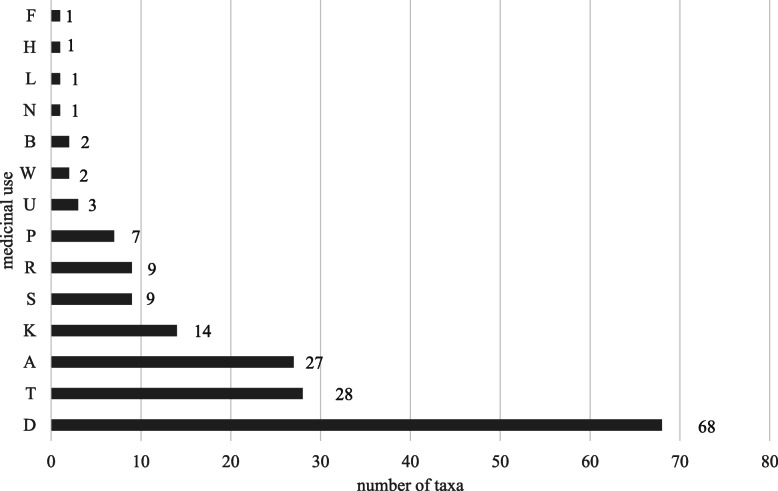
Taxa frequencies of the vegetables for local medicinal uses. Notes: A: general and unspecified; B: blood, blood-forming organs and immune mechanism; D: digestive, F: eye, H: ear, K: cardiovascular, L: musculoskeletal, N: neurological, P: psychological, R: respiratory, S: skin, T: endocrine/metabolic and nutritional, U: urological, W: pregnancy/childbearing, family planning

Many of the vegetables were cited as functional food with health benefits by the informants (106 taxa, 80.3%), while others were regarded as just “good for health” (26 taxa, 19.7%). The traditional knowledge on the treatment of ailments is listed in Additional file 1, Appendix B. The vegetables cited were used mainly to treat dyspepsia/indigestion (33 taxa), vitamin/nutritional deficiency (18 taxa), diarrhoea (15 taxa) and general weakness/tiredness (8 taxa) (Fig. 3).

**Fig. 4:**
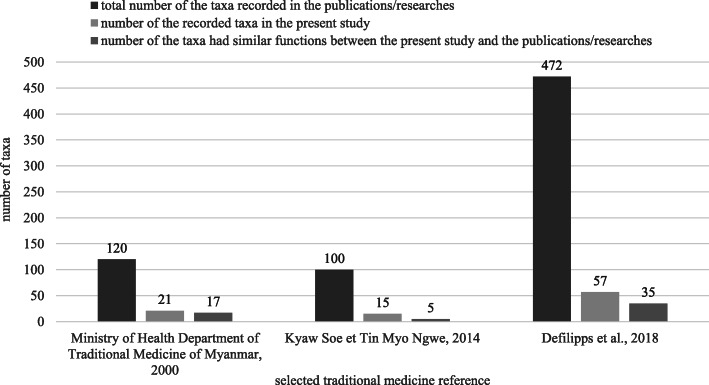
Bibliography comparison: the vegetables recorded as traditional Myanmar medicinal plants in the selected literature

Sixty-four taxa were recorded as traditional Myanmar medicinal plants in the selected literature on traditional Myanmar medicine, [16, 47, 48]. In these publications and studies, 57 taxa were recorded in [16], 21 in [48] and 15 in [47] (Additional file 1, Appendix B). Comparing the local health knowledge recorded in the present study and the records of these previous selected publications and studies, the local health knowledge of 35 taxa was similar to the records in [16], 17 taxa in [48] and five taxa in [47] (Fig. 4). Nevertheless, forty-seven taxa were not recorded in the three selected studies but were still used as healthy vegetables by local people.

## Discussion

### Rich diversity of vegetable plants

We found a rich diversity of vegetable resources covering a broad range of genera and families from the markets of central Myanmar. In the present study, 132 plant taxa were documented (Table 2). Compared with previous studies in the Indo-Burma biodiversity hotspot region, central Myanmar was relatively high in terms of the diversity of plants used as vegetables. For example, in Yunnan Province, China, 68 species of wild plants were used as healthy vegetables by native people of Wa, Blang, Lahu and Dai ethnic nationalities [49]. In Chiang Mai Province, Thailand, 94 woody plants, such as mango and fig species, were cultivated for vegetables, fruits and other uses in home gardens [6]. In the Koch Bihar District of West Bengal, India, 125 wild species were recorded as edible plants [50]. Sixty-eight wild species were traditionally consumed in the Manipur district of India [37].

Twenty-six taxa were assessed by The International Union for Conservation of Nature. Only one species (*Kaempferia candida*) was designated an endangered species at the vulnerable (VU) level, while others were not endangered. The only endangered species was noted only twice (UR = 2) by two isolated informants. The informants claimed that they cultivated the plant in their home gardens.

### Wild vegetable resources

The most frequently cited vegetables commonly appeared elsewhere in the region and were noted as “staple goods”, which reflects the fact that large-scale cultivation comprises the major source of vegetables for the urban population of central Myanmar. However, twenty-eight species of the vegetables we recorded were collected from wild places.

In the present study, we found that it was difficult to divide native vegetable species into “wild vegetables” and “cultivated vegetables” because most of the plants were collected not only from wild places but also from home gardens and vegetable fields. Some local vegetable growers told us that the reason they cultivated them was that these vegetables sold well in markets, but the limited wild resources could not meet the demand of markets. In the field survey, interestingly, most of the interviewed vegetable sellers claimed that their goods were from the wild, possibly because wild vegetables were more expensive than cultivated vegetables in the markets. To verify the actual situation, we visited local home gardens and vegetable fields in both urban and rural areas. Finally, just twenty-eight species of the vegetables we recorded were actually all from wild sources, while others were cultivated more or less by local people.

It seemed that the diversity of wild vegetables was relatively low (28 species, 21% of the total) in central Myanmar according to the results of the present study. A possible reason was that we counted only the vegetables that were entirely from wild sources as “wild vegetables” in the field study. Another possible reason was that the quite dry climate limits the wild plant diversity of central Myanmar. However, we do not have enough data to verify this possibility at present. Therefore, we will focus on the plant species diversity correlation between the wild places and local markets in our future follow-up studies.

### Important vegetables and their health functions

**Fig. 5:**
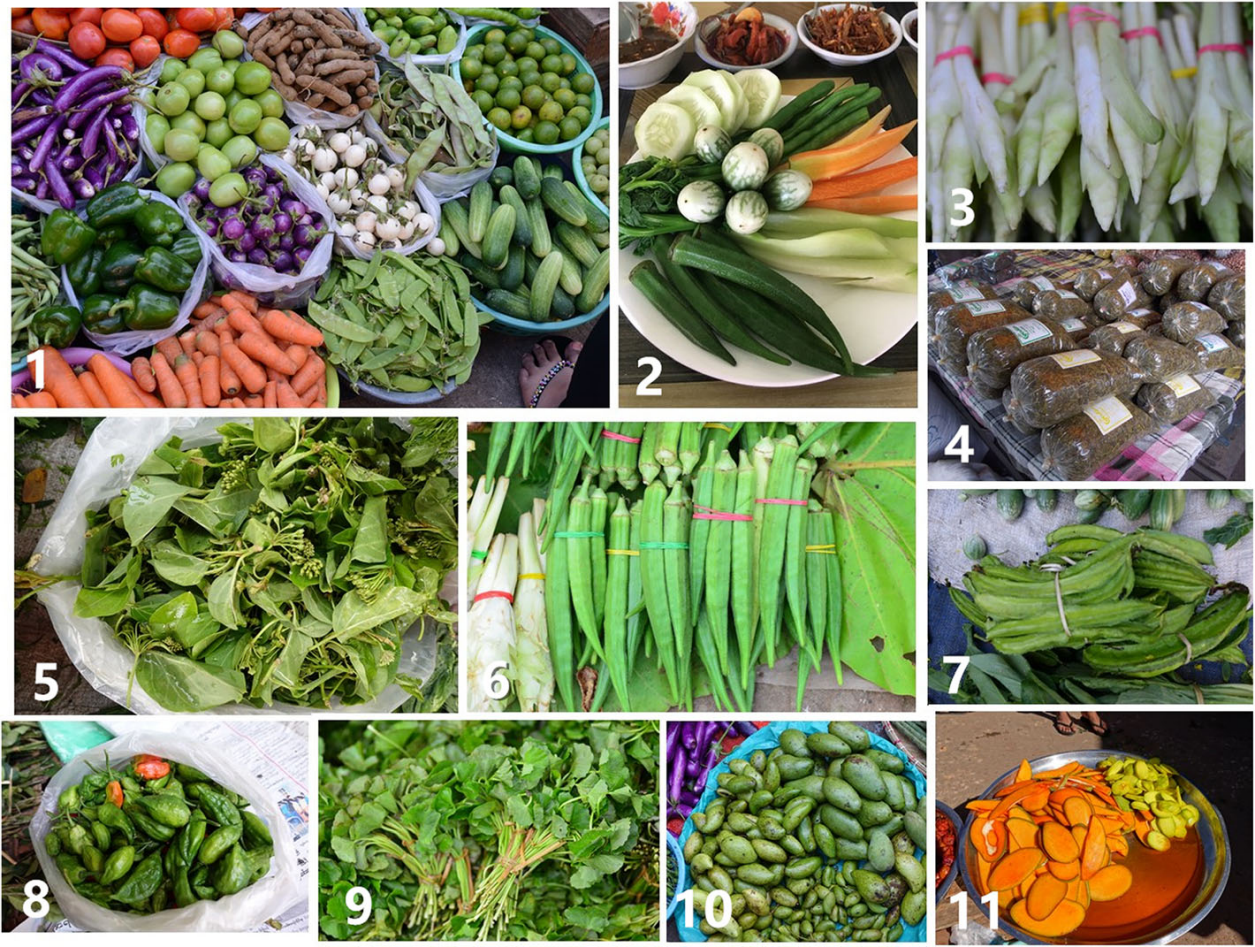
Rich diversity of vegetables and typical important vegetables in local markets. Note: 1: A vegetable stall. 2: Typical mixed vegetable salad dish of the local daily cuisine. 3: Inflorescences of *Curcuma longa*. 4: Fermented tea leaf. 5: *Dregea volubilis*. 6: *Abelmoschus esculentus*. 7: *Psophocarpus tetragonolobus*. 8: *Capsicum annuum*. 9: *Centella asiatica.* 10: Fresh mango. 11: Mango salad

The results of the quantitative analysis indicate that some of the recorded plants were important for local people. These plants were used repeatedly in the local daily cuisine, and the local people claimed that some of them had health functions (Fig. 5).

As indicated by the UV and RFC values, mango (*Mangifera indica*) was the most cited vegetable in the present study. As a native species [51], *Mangifera indica* has been used by Myanmar people for a long time [52] and in multiple ways. A well-known proverb states that “Mango us the best fruit and tea leaf is the best leaf” [53]. Mango leaves are used as medicine to treat diarrhoea, and the young sour fruits are made into salad, while mature sweet fruits are one of the most famous tropical fruits.

Fermented tea leaf (La-phet) is a unique vegetable that we found in Myanmar [53]. It is a typical traditional ingredient in the daily cuisine. It was considered to be beneficial to digestion by the informants. The main species used is *Camellia sinensis* var. *assamica*, which is identical to pu’er tea in Chinese tea. Fermented tea leaf is also enjoyed as a daily vegetable by the local ethnic people in Yunnan Province, China, North Thailand and Laos [54]. Tea leaves were commonly used as a vegetable in ancient Asia [55]. The history of cooking tea leaves in Asia can be traced to 2500 years ago from the history, literature and tomb relics of ancient China. Chinese people cooked tea leaves in soup, pickles and congee in the Zhou, Qin and Han Dynasties [55].

Chili (*Capsicum annuum*) is an important ingredient in Myanmar cuisines as well as in the cuisines of neighbouring countries. The uses of *C. annuum* in South and Southeast Asia can be traced to 500 years ago, when Portuguese merchants introduced the species to India [56]. Many cultivars were sold in the markets, and the appearance and taste vary. *C. annuum* is a common medicinal plant in traditional Myanmar medicine [16]. It is used by local people to treat colds and digestive problems.

Okra (*Abelmoschus esculentus*), originating from Africa and spreading to almost all tropical and sub-tropical regions [57, 58], is very common in the daily diet of Myanmar and is used to treat diabetes. Water spinach (*Ipomoea aquatic*) is one of the most popular vegetables in Myanmar and is usually cooked with mushrooms, as water spinach is a powerful antidote to poisonous mushrooms, according to the informants.

Fabaceae is the most commonly used family, with 17 species used by local people. They are cultivated mainly as a source of protein, which is contained mainly in the seeds. Apart from seeds, local people also collect the tender leaves, shoots, flowers and fleshy roots as vegetables. For example, the tender shoots, fruits and fleshy roots of *Psophocarpus tetragonolobus* are collected as vegetables in different seasons by local people. In traditional Myanmar medicine, tender fruits are used to treat diabetes, while leaves are used to treat eye diseases and toothache. Previous studies have shown that the leaves of this species are rich in protein and vitamins A and C [59].

The Zingiberaceae species are also important components of the local cuisine. Eight Zingiberaceae species were recorded in the present study, with *Curcuma longa* (UR = 80) the most frequently occurring species. *C. longa* is also known as turmeric in English and is one of the most famous medicinal plants produced in tropical areas. It can be used to treat digestive problems, according to the informants. Curcumin, a polyphenol that has diverse anti-inflammatory and anti-cancer properties, is the functional ingredient of the species and is usually administered orally or topically [9].

Among the taxa recorded, 57 species were also noted as medicinal plants by Defilipps and Krupnick [16], of which 35 species had functions similar to those noted in the results of our study. Likewise, 22 species and 15 species were found in common with the herbal book edited by the Ministry of Health Department of Traditional Medicine of Myanmar in 2000 [48] and with the results of Kyaw Soe and Tin Myo Ngwe in 2014 [47], respectively. All of the vegetables collected from the wild are used for both food and traditional medicine [16, 47, 48]. These species are common in the natural vegetation of central Myanmar. In traditional Myanmar medicine, *Dregea volubilis*, the most commonly used wild species, is used to treat multiple diseases, such as indigestion, dyspepsia, dysentery, diarrhoea and insomnia [59]. The functional compounds of the species are a group of dregeosides that have effective anti-bacterial, anti-oxidant and anti-diabetic properties [60–62]. *Centella asiatica* can treat symptoms of insomnia and forgetfulness, according to the informants. In traditional Myanmar medicine, *C. asiatica* is used to treat memory impairment, oliguria and eye diseases [59]. A modern study found that an extract of the species had effective anti-Alzheimer’s disease properties [63].

Even the most common vegetables worldwide, such as *Solanum melongena* and *Brassica rapa*, which had the highest RFC values in the present study, are used in particular ways for health care by local people. The informants claimed that *S. melongena* is a good tonic for treating weakness, and *B. rapa* is used to treat cancer and skin infection (external use). Previous studies have shown that these vegetables contain some functional compounds with effective properties for human health [64–66]. For example, glucosinolates, a group of effective compounds found in *Brassica rapa*, have many potential functions, such as anti-microbial, anti-cancer and anti-parasite properties [67]. Nevertheless, there is still no clinical evidence to prove the anti-cancer properties of these compounds in the human body.

### Lesser known vegetables

In the present study, we found that the most frequently cited vegetables commonly appeared elsewhere in the region and were noted as “staple goods”. According to the results of quantitative analysis, these plants were designated “the most popular vegetables” in the local markets. However, this could lead us to ignore the lesser known vegetables, which reflect the unique food culture of local people. Most of these lesser known vegetables were cited only a few times by the informants, which caused them to rank low in the league table of UV and RFC values.

Some of these lesser known vegetables, such as *Curcuma glauca*, *Terminthia paniculata* and *Ottelia cordata*, were cited by only one informant. *Curcuma glauca* seemed to be distributed only in central Myanmar, according to a previous study [68, 69]. This species was sold by only one informant in Nay Pyi Taw, and it may be available only in mid-August. The inflorescences of the species were used as a spice in salads. *Terminthia paniculata* is a typical wild species of the dry, hot region of tropical Asia [8]. An informant used the fruits of the species in sour soup cooking. *Ottelia cordata* appeared only in December and was sold by one informant in Mandalay.

Some species are common plants but are used in unique ways by local people, such as the fermented tea leaf mentioned above. *Aloe vera* is a well-known skin-care herbal plant, but local people cook the leaves of the species for Myanmar-style salads. Similarly, *Caralluma edulis* is an important ingredient of local-style curries in Mandalay, while it is a popular ornamental succulent in many tropical gardens and greenhouses. The leaves of *Ficus carica* were sold as flavour by one informant in Mandalay because of the special “coconut fragrance” of these leaves, and the species is popular as fruit trees that were cultivated widely. Even some well-known “weeds”, such as *Ludwigia adscendens*, *Enydra fluctuans* and *Eclipta prostrata*, are cooked as delicacies by local people.

Forty-seven taxa (35.6% of the total) are used as healthy vegetables by local people, although they were not included in the selected herbal books. Future revisions of traditional Myanmar medicine books should pay more attention to these plants.

### Issues of food safety

Food safety is always an important topic. In the present study, some species found in the market were considered to be possibly poisonous or carcinogenic, such as *Colocasia esculenta*, *Archidendron pauciflorum*, *Azadirachta indica*, *Solanum torvum*, *Manihot esculenta* and some Apocynaceae species. The informants were aware of the risk of consuming these species, and local knowledge was available on the preparation and treatment of the raw plant materials before consumption. The preparation includes blanching in boiling water, stewing for a long time, boiling with detoxification materials and salting. Some previous studies in food chemistry have shown that these preparation methods can help to reduce toxins to a certain degree [65].

For example, the corms of *Colocasia esculenta* are rich in needle-like calcium oxalate crystals that can heavily irritate oral and respiratory mucosa, but boiling for a long time at a high temperature can reduce the irritation. High-temperature cooking can also degrade the nephrotoxic compounds in *Archidendron pauciflorum*. For *Azadirachta indica*, the tender shoots and leaves should be boiled with sour material such as tamarinds until the bitter taste becomes reduced and then made into salads and soups because the bitter compounds of the species are regarded as toxic [9].

However, local knowledge alone would not be sufficient for food safety management, and modern management approaches to regulating and monitoring these poisonous vegetables and public awareness and education based on scientific research are fundamentally needed. Future research could focus on surveying and recording local knowledge of food safety and attempt to establish vegetable food safety management standards based on local knowledge.

## Conclusion

The diversity and use of the healthy vegetables in central Myanmar were very rich. The most frequently occurring species were *Capsicum annuum*, *Solanum melongena*, *Brassica rapa*, *Camellia sinensis* var. *assamica* and *Ipomoea aquatica*, while *Mangifera indica*, *Capsicum annuum*, *Abelmoschus esculentus*, *Ipomoea aquatic* and *Cucumis sativus* had the richest uses and knowledge. Only one species (*Kaempferia candida*) was designated VU by the IUCN but was not used frequently.

The most frequently cited vegetables were commonly cultivated species, and just twenty-eight species of the vegetables we recorded were actually all from wild sources, while others were more or less cultivated by local people. Lesser known vegetables could reflect the unique food culture of local people. However, most of them were ignored because of their low ranking in the league table of UV and RFC values because most of them were cited only a few times by the interviewees.

Most of these vegetables were also recorded in the selected traditional Myanmar medicine books. Nevertheless, some taxa were used as healthy vegetables by local people but not recorded in these books. Future revisions of traditional Myanmar medicine books should pay more attention to these plants.

In the present study, some species were poisonous, and local people had knowledge of ways to reduce toxins. However, from the perspective of public health, depending only on local knowledge to treat food safety problems of healthy vegetables is not enough. Future research should focus on documenting and managing local food safety knowledge about these vegetables.

## Supplementary Information

**Additional file 1:** Appendix A: Table 1 Wild vegetables. Appendix B: Table 2 Plant list

## Data Availability

Please contact the author for data requests.
